# Sarcopenia as a potential risk factor for senile blepharoptosis: Nationwide Surveys (KNHANES 2008–2011)

**DOI:** 10.1038/s41598-023-31097-7

**Published:** 2023-03-29

**Authors:** Hyeong Ju Byeon, Yong Joon Kim, Jin Sook Yoon, JaeSang Ko

**Affiliations:** Department of Ophthalmology, Severance Hospital, Institute of Vision Research, Yonsei University College of Medicine, 50-1 Yonsei-Ro, Seodaemun-Gu, Seoul, 03722 South Korea

**Keywords:** Medical research, Pathogenesis, Risk factors

## Abstract

As the world’s population is aging, sarcopenia is recognized as essential to assess people’s lifelong condition and do appropriate early intervention. Senile blepharoptosis is also a problem in old age deteriorating visual function and causing a cosmetic decline. We investigated the association between sarcopenia and the prevalence of senile blepharoptosis, using a nationwide representative survey in Korea. A total of 11,533 participants were recruited. We used the body mass index (BMI)- adjusted appendicular skeletal muscle (ASM) definition as the muscle mass index (MMI, ASM [kg] divided by BMI [kg/m^2^]). The association between blepharoptosis prevalence and MMI was analyzed using multivariate logistic regression. Sarcopenia, defined as the lowest MMI quintile group in both men and women, was also associated with the prevalence of blepharoptosis (ORs 1.92, 95% CI 1.17–2.16; p < 0.001). These associations remained statistically significant after adjusting for various factors related to blepharoptosis using multivariate analysis (ORs 1.18, 95% CI 1.04–1.34; p = 0.012). Moreover, MMI was found to have a proportional relationship with eyelid lifting force (levator function), which is closely related to the occurrence and severity of ptosis. Sarcopenia is related to the prevalence of senile blepharoptosis, and patients with lower MMI were more likely to have blepharoptosis. These results suggest that sarcopenia can affect visual function and aesthetics.

## Introduction

Sarcopenia was first described as an age-related decline in the lean body mass. However, as the world’s population is aging and sarcopenia is recognized to start earlier in life^1,2^, assessing people’s lifelong condition and appropriate early intervention has become essential. In addition, many researchers have reported that untreated sarcopenia impairs daily living, lowers the quality of life, and imposes burdens on the personal and social economy^3^. This is reflected in the introduction of sarcopenia into the International Classification of Diseases-10 codes in 2016^4–6^. Moreover, this concept was extended to include muscle function, represented as muscle strength and physical performance, in 2018, led by the European Working Group on Sarcopenia in Older People 2 (EWGSOP2)^7^.

Blepharoptosis is defined as drooping of the upper eyelid, which impairs quality of life by blocking the visual axis and causing cosmetic concerns^8,9^. Based on etiology, it is classified as aponeurotic, myogenic, neurogenic, mechanical, or traumatic^10^. Acquired blepharoptosis in old age is called senile blepharoptosis. As senile blepharoptosis is characteristic of disinserted levator aponeurosis from the upper eyelid tarsal plate, which cannot effectively transfer muscle contraction, it was also considered aponeurotic blepharoptosis. However, except for the aponeurotic factor, previous studies have reported that senile blepharoptosis shows degenerative changes of muscle itself including fat infiltration^11–14^. Levator palpebrae superioris muscle is also a skeletal muscle, and sarcopenia, a progressive and generalized skeletal muscle disorder, is expected to be associated with blepharoptosis. However, there are no reports on the relationship between sarcopenia and blepharoptosis. In this study, we investigated the association between sarcopenia and senile blepharoptosis using a nationwide representative survey in Korea.

## Results

### Baseline characteristics

Finally, 11,553 participants (5159 men and 6394 women) aged 40–79 years who completed the ophthalmological examination and DXA were included (Table 1). The mean age of the group with blepharoptosis was 62.8 ± 10.3 years in men and 65.3 ± 9.4 years in women, which is significantly older than the group without blepharoptosis (56.4 ± 10.8 years in men, 56.0 ± 10.7 years in women, *p* < 0.001). Mean MMI was significantly lower in individuals with blepharoptosis than those without blepharoptosis in both men and women (0.86 ± 0.10 (kg/kg/m^2^), 0.90 ± 0.11 (kg/kg/m^2^) in men, with and without blepharoptosis respectively; *p* < 0.001, 0.57 ± 0.08 (kg/kg/m^2^), 0.60 ± 0.08 (kg/kg/m^2^) in women, with and without blepharoptosis respectively; *p* < 0.001).

**Table 1:** Baseline characteristics of the study population.

	Men (N = 5159)		p	Women (N = 6394)		p
	Blepharoptosis (N = 781)	No blepharoptosis (N = 4378)		Blepharoptosis (N = 918)	No blepharoptosis (N = 5476)	
Age (years) (range)	62.8 ± 10.3 (40–79)	56.4 ± 10.8 (40–79)	< 0.001	65.3 ± 9.4 (40–79)	56.0 ± 10.7 (40–79)	< 0.001
Age groups			< 0.001			< 0.001
40–49, n (%)	112 (14.3%)	1416 (32.3%)		66 (7.2%)	1827 (33.4%)	
50–59, n (%)	163 (20.9%)	1221 (27.9%)		166 (18.1%)	1617 (29.5%)	
60–69, n (%)	250 (32.0%)	1093 (25.0%)		321 (35.0%)	1248 (22.8%)	
70–79, n (%)	256 (32.8%)	648 (14.8%)		365 (39.8%)	784 (14.3%)	
Hypertension, n (%)	308 (39.4%)	1249 (28.5%)	< 0.001	412 (44.9%)	1500 (27.4%)	< 0.001
Diabetes, n (%)	148 (18.8%)	508 (11.6%)	< 0.001	159 (17.3%)	484 (8.8%)	< 0.001
Dyslipidemia, n (%)	99 (12.7%)	480 (11.0%)	0.182	141 (15.4%)	760 (13.9%)	0.253
Stroke, n (%)	36 (4.6%)	130 (3.0%)	0.022	38 (4.1%)	111 (2.0%)	< 0.001
Ischemic heart disease, n (%)	40 (5.1%)	155 (3.5%)	0.042	35 (3.8%)	163 (3.0%)	0.211
Smoking, n (%)	280 (35.9%)	1702 (39.0%)	0.12	38 (4.2%)	249 (4.6%)	0.64
Regular exercise, n (%)	114 (14.6%)	830 (19.0%)	0.004	111 (12.1%)	767 (14.1%)	0.131
Height (cm)	166.4 ± 5.8	168.1 ± 6.1	< 0.001	152.3 ± 6.0	155.1 ± 5.9	< 0.001
Body weight (kg)	66.1 ± 9.8	67.1 ± 10.0	0.014	56.5 ± 9.0	57.2 ± 8.5	0.029
Fat mass (kg)	15.5 ± 5.0	15.1 ± 4.9	0.018	19.7 ± 5.6	19.5 ± 5.2	0.318
Lean body mass (kg)	50.6 ± 6.5	52.0 ± 6.7	< 0.001	36.8 ± 4.7	37.7 ± 4.7	< 0.001
ASM (kg)	20.6 ± 3.0	21.5 ± 3.2	< 0.001	14.0 ± 2.0	14.4 ± 2.1	< 0.001
Fat percentage (%)	23.1 ± 5.3	22.1 ± 5.1	< 0.001	34.3 ± 5.7	33.6 ± 5.3	0.001
WC (cm)	86.5 ± 9.0	85.0 ± 8.5	< 0.001	83.6 ± 9.4	80.7 ± 9.3	< 0.001
BMI (kg/m^2^)	23.8 ± 3.0	23.7 ± 2.9	0.266	24.3 ± 3.3	23.8 ± 3.2	< 0.001
FMI (kg/m^2^)	5.6 ± 1.8	5.3 ± 1.7	< 0.001	8.5 ± 2.3	8.1 ± 2.1	< 0.001
LBMI (kg/m^2^)	18.2 ± 1.8	18.4 ± 1.8	0.048	15.9 ± 1.5	15.7 ± 1.6	0.001
MMI (kg/kg/m^2^)	0.86 ± 0.10	0.90 ± 0.11	< 0.001	0.57 ± 0.08	0.60 ± 0.08	< 0.001
MMI quintile groups			< 0.001			< 0.001
Q1, lowest, n (%)	238 (29.2%)	804 (18.4%)		283 (30.8%)	996 (18.2%)	
Q2, n (%)	192 (24.6%)	840 (19.2%)		200 (21.8%)	1079 (19.7%)	
Q3, n (%)	147 (18.8%)	885 (20.2%)		168 (18.3%)	1111 (20.3%)	
Q4, n (%)	123 (15.7%)	909 (20.8%)		154 (16.8%)	1125 (20.5%)	
Q5, highest, n (%)	91 (11.7%)	940 (21.5%)		113 (12.3%)	1165 (21.3%)	
Levator function			< 0.001			< 0.001
Good (≥ 12 mm), n (%)	244 (31.2%)	2934 (67.0%)		245 (26.7%)	3509 (64.1%)	
Fair (8–11 mm), n (%)	398 (51.0%)	1325 (30.3%)		450 (49.0%)	1796 (32.8%)	
Poor (≤ 7 mm), n (%)	139 (17.8%)	119 (2.7%)		223 (24.3%)	171 (3.1%)	
Lens			< 0.001			< 0.001
Cataract, n (%)	427 (55.0%)	1586 (36.3%)		540 (59.1%)	1915 (35.0%)	
No cataract, n (%)	280 (36.0%)	2654 (58.7%)		256 (28.0%)	3239 (59.2%)	
Pseudophakia, n (%)	65 (8.4%)	218 (5.0%)		117 (12.8%)	312 (5.7%)	
Aphakia, n (%)	5 (0.6%)	3 (0.1%)		1 (0.1%)	3 (0.1%)	

*ASM* Appendicular skeletal muscle mass, *WC* Waist circumference, *BMI* Body mass index, *FMI* Fat mass index, *LBMI* Lean body mass index, *MMI* Muscle mass index.

Participants of both sexes with blepharoptosis had a higher prevalence of hypertension and diabetes (p < 0.001). Women with blepharoptosis had a higher BMI (p < 0.001); however, there was no difference in BMI between men with and without blepharoptosis (*p* = 0.266).

### Muscle mass index and blepharoptosis

**Figure 1:**
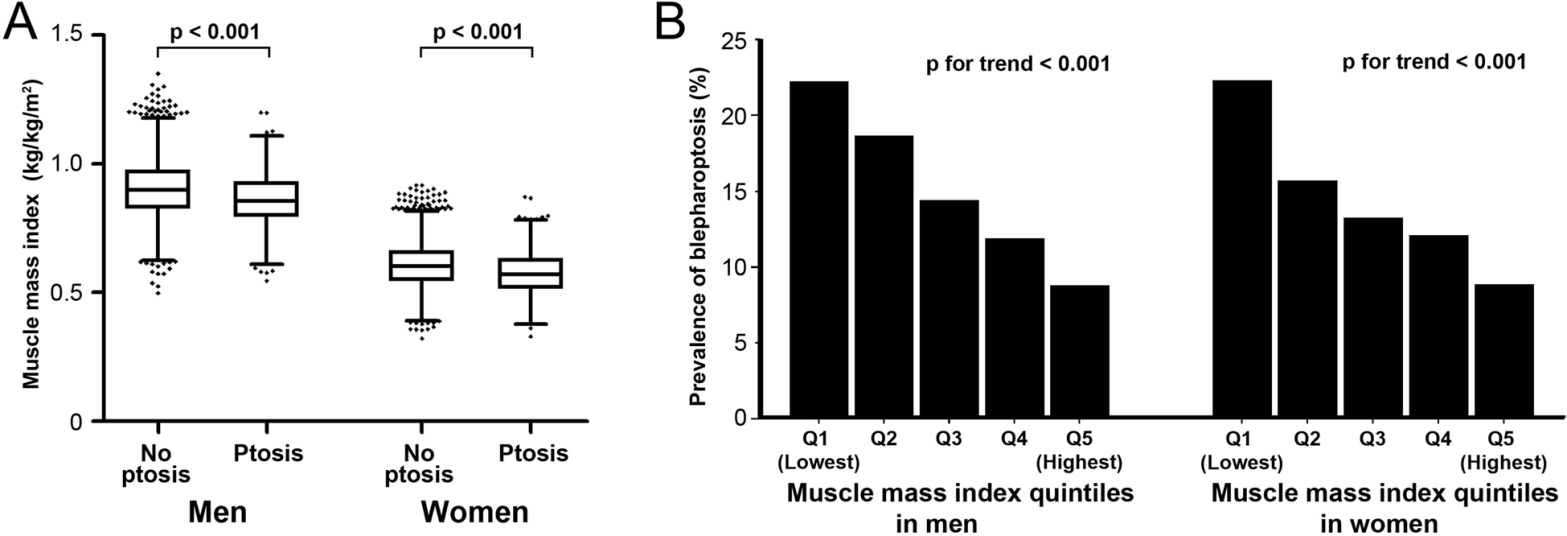
Muscle mass index and blepharoptosis prevalence. (**A**) The muscle mass index distribution is lower in the blepharoptosis group compared to the no blepharoptosis group (*p* < 0.001). (**B**) The prevalence of blepharoptosis increased as MMI decreased (*p* value for linear trend < 0.001).

The calculated MMI ranged from 0.498 to 1.350 (kg/kg/m^2^) and from 0.322 to 0.917 (kg/kg/m^2^) in men and women, respectively (Fig. 1A). Individuals in the lowest MMI quintile group in men and women were defined as having sarcopenia, and the cutoff values were 0.809 kg/kg/m^2^ in men and 0.532 kg/kg/m^2^ in women. A negative correlation was found between MMI and blepharoptosis prevalence; the lower the MMI, the higher the prevalence of blepharoptosis (Fig. 1B, p value for linear trend < 0.001 in both men and women). This trend of negative correlation between MMI and blepharoptosis was also maintained when stratified with ages 40–59 and 60–79 (Supplementary Fig. S1).

Using multivariable logistic regression, the effects of potential confounders (age, hypertension, diabetes, obesity, smoking, and history of cataract surgery) were adjusted to evaluate the association between MMI and blepharoptosis (Table 2). The degree of MMI affecting blepharoptosis, represented as an odds ratio (OR), was greater in men than in women. In men, after adjusting for all potential confounding factors in model 3, the prevalence of blepharoptosis had a significant inverse association with MMI (OR, 0.17; 95% CI 0.07–0.39; *p* < 0.001). In women, similar to men, a significant negative association between MMI and the prevalence of blepharoptosis was found in model 2 (OR, 0.36; 95% CI 0.13–0.96; *p* = 0.042).

**Table 2:** Multivariate analysis of muscle mass index and blepharoptosis prevalence.

	Crude	Model 1	Model 2	Model 3
Muscle mass index				
Men				
OR	0.02	0.12	0.13	0.17
95% CI	(0.01, 0.05)	(0.05, 0.27)	(0.06, 0.31)	(0.07, 0.39)
* p-*value	< 0.001	< 0.001	< 0.001	< 0.001
Women				
OR	0.01	0.31	0.36	0.40
95% CI	(0.00, 0.02)	(0.12, 0.81)	(0.13, 0.96)	(0.14, 1.14)
* p-*value	< 0.001	0.017	0.042	0.086

*OR* Odds ratio.

Model 1: adjusted for age.

Model 2: adjusted for age, hypertension, diabetes, smoking, and history of cataract surgery.

Model 3: adjusted for age, hypertension, diabetes, smoking, history of cataract surgery, and obesity (BMI > 25).

### Sarcopenia and blepharoptosis

After identifying a linear correlation between MMI and blepharoptosis prevalence, the relationship between sarcopenia, defined as the lowest MMI quintile group, and blepharoptosis prevalence was analyzed using a multivariate analysis (Table 3). Even after adjusting for multiple confounders (age, sex, hypertension, diabetes, history of cataract surgery, and obesity), sarcopenia was significantly correlated with the prevalence of blepharoptosis (OR, 1.18; 95% CI 1.04–1.34; *p* = 0.012).

**Table 3:** Multivariate analysis of sarcopenia and blepharoptosis prevalence stratified by levator function.

	Crude	Model 1	Model 2	Model 3
Total population (n = 11,553)				
Sarcopenia*				
OR	1.92	1.25	1.23	1.18
95% CI	(1.71, 2.16)	(1.10, 1.41)	(1.08, 1.39)	(1.04, 1.34)
* p-*value	< 0.001	< 0.001	0.001	0.012
Men^†^ (n = 5159)				
Sarcopenia*				
OR	1.83	1.30	1.28	1.22
95% CI	(1.54, 2.18)	(1.09, 1.56)	(1.06, 1.54)	(1.01, 1.47)
* p*-value	< 0.001	0.004	0.009	0.038
Women^†^ (n = 6394)				
Sarcopenia*				
OR	2.00	1.20	1.19	1.17
95% CI	(1.72, 2.34)	(1.01, 1.42)	(1.00, 1.41)	(0.98, 1.39)
* p*-value	< 0.001	0.034	0.048	0.085
LF ≥ 8 mm (n = 10,901)				
Sarcopenia*				
OR	1.88	1.26	1.24	1.19
95% CI	(1.66, 2.14)	(1.10, 1.44)	(1.08, 1.43)	(1.04, 1.37)
* p-*value	< 0.001	< 0.001	0.002	0.013
LF ≤ 7 mm (n = 652)				
Sarcopenia*				
OR	1.18	0.96	0.95	1.00
95% CI	(0.80, 1.74)	(0.64, 1.43)	(0.63, 1.44)	(0.65, 1.53)
* p-*value	0.402	0.836	0.820	0.992

*LF* Levator function, *OR* Odds ratio.

Model 1: adjusted for age and sex.

Model 2: adjusted for age, sex, hypertension, diabetes, smoking, and history of cataract surgery.

Model 3: adjusted for age, sex, hypertension, diabetes, smoking, history of cataract surgery, and obesity (BMI > 25).

*Individuals in the lowest MMI quintile group in men and women were defined as having sarcopenia.

^†^All models were adjusted for the same multivariates except sex.

### Sarcopenia, blepharoptosis, and levator function

**Figure 2:**
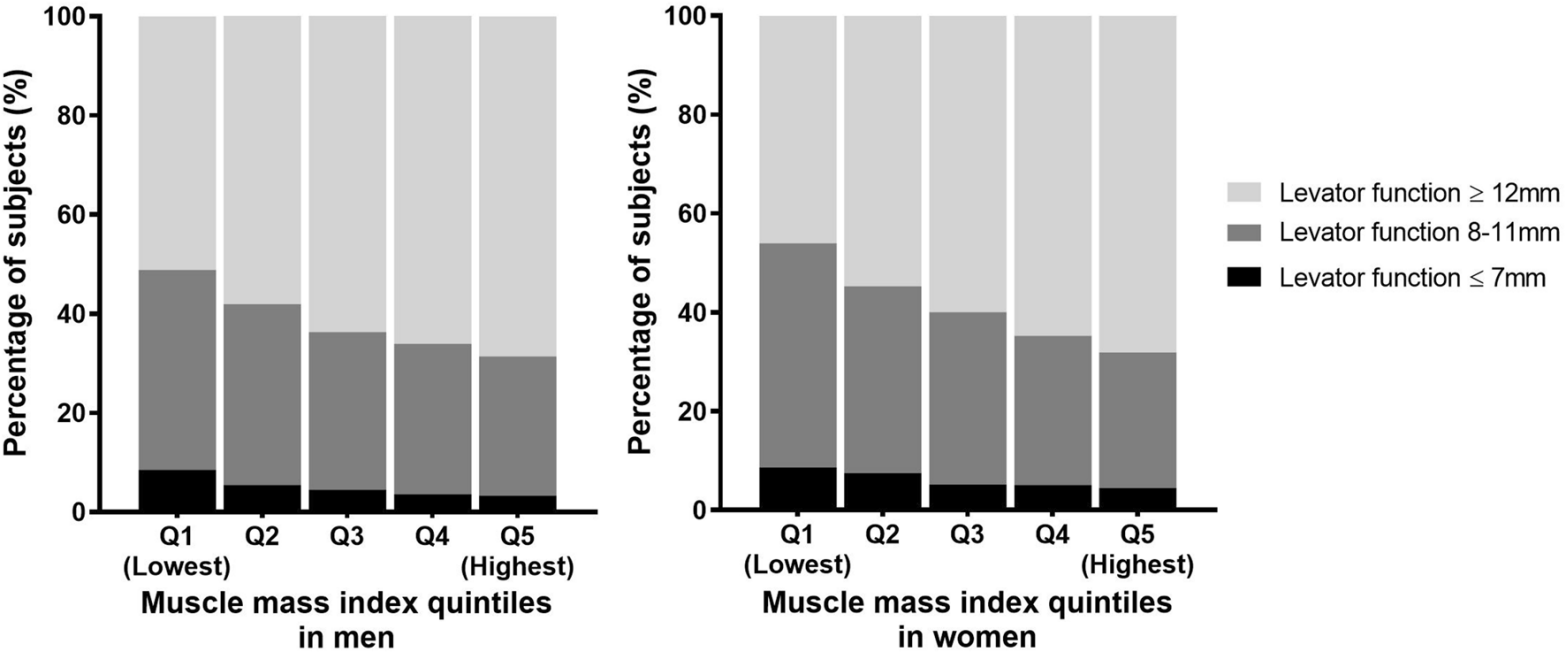
Muscle mass index and blepharoptosis stratified with levator function. As muscle mass index decreased, the distribution of participants with poor levator function increased (all *p* value for linear trend in each levator function groups < 0.001).

Levator function was compared according to MMI quintile groups. Participants with lower MMI showed decreased levator function, indicating a linear correlation between appendicular muscle mass and eyelid lifting force. (Fig. 2; all *p* values for linear trend in each levator function classification < 0.001).

Patients were stratified according to levator function, and the relationship between sarcopenia and blepharoptosis prevalence was analyzed using multivariate logistic regression (Table 3). In the fair to good levator function group (levator function ≥ 8mm), the correlation between blepharoptosis and sarcopenia, which was found in the entire population, was also identified (OR, 1.19; 95% CI 1.04–1.37; *p* = 0.013). However, there was no significant relationship between sarcopenia and blepahroptosis in the poor levator function group (levator function ≤ 7mm) (OR, 1.00; 95% CI 0.65–1.53; *p* = 0.992).

### BMI and blepharoptosis

Since previous studies have reported obesity as a potential risk factor for blepharoptosis^16,17^, the relationship between BMI and the prevalence of blepharoptosis was analyzed using the same methods as in this study. Divided by BMI quintiles, the incidence of blepharoptosis had no relation with BMI in men (*p* value for linear trend = 0.359) but a positive relationship in women (*p* value for linear trend < 0.001) (Supplementary Fig. S2A).

Levator function (good, ≥ 12 mm; fair, 8–11 mm; poor, ≤ 7 mm) in each BMI quintile group was analyzed (Supplementary Fig. S2B). Women with high BMI showed significantly low levator function (all *p* values for linear trend in each levator function group < 0.05), but the relevance was not as evident as MMI in Fig. 2. Moreover, men had no relationship between BMI and levator function (all *p* values for linear trend in each levator function group > 0.05).

## Discussion

This study demonstrated an association between sarcopenia and blepharoptosis. The sarcopenia representative index, MMI, was significantly inversely correlated with the prevalence of blepharoptosis, and MMI was an independent variable after adjusting for age, sex, hypertension, diabetes, smoking, cataract surgery history, and obesity. The levator function of the eyelid was also significantly associated with MMI, and we could assume that sarcopenic patients with low MMI showed lower levator function, causing blepharoptosis.

**Figure 3:**
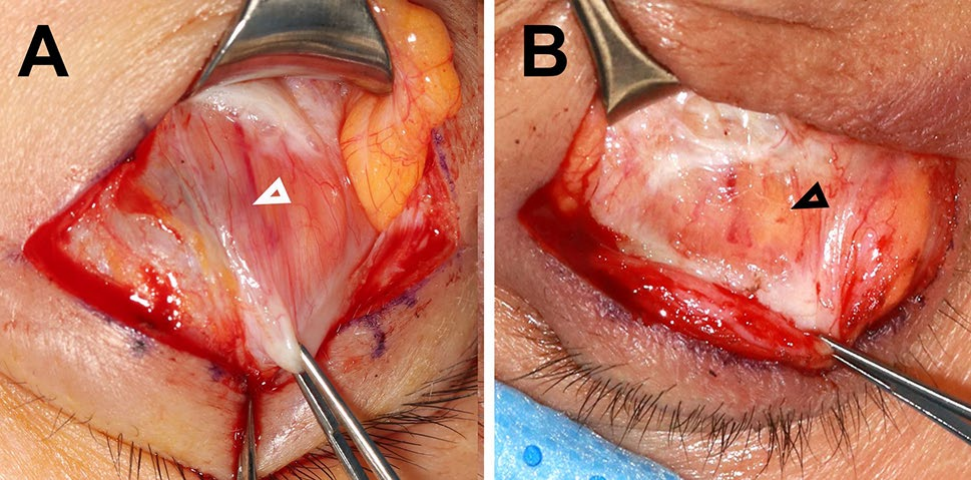
Intraoperative findings of levator palpebrae superioris muscle. These pictures were taken by one of the authors (J.K.) during routine blepharoplasty surgery. (**A**) The white arrowhead indicates normal levator palpebrae superioris muscle showing muscle fibers. (**B**) The black arrowhead indicates fat infiltration of the levator palpebrae superioris muscle.

Sarcopenic muscles exhibit two apparent changes as part of the aging process. The first is increased intramuscular fatty infiltration^6,18,19^. Inter and intramuscular lipid accumulation is accelerated with aging, as fibro-adipogenic precursor cells reside in muscular tissue and differentiate into adipocytes. In addition, intramyocellular lipid droplets are mediated by impaired hormone regulation and mitochondrial dysfunction during aging^20^. Fat infiltration into the muscle is not only a burden for muscle activity but also a preliminary step in infiltrating macrophages, inflammatory cytokines, and fat-associated hormones^21^. Likewise, on general intraoperative observations, the levator palpebrae superioris muscle in involutional blepharoptosis patients frequently shows myosteatosis (Fig. 3). Previous reports have shown that the group with higher fat infiltration had lower levator muscle function, consequently resulting in blepharoptosis^11,22–24^. Second, the loss of motor units is accelerated in type II muscle fibers, and additional atrophy of type II muscle fibers decreases muscle mass and gradually decreases muscle power^6,19^. The levator palpebrae superioris muscle is also composed of type I and II muscle fibers, approximately 85% of which are type II^25^. With a high proportion of type II muscle fibers, these sarcopenic changes can also occur in the levator palpebral superior muscle and deteriorate its function.

Several studies have reported that blepharoptosis is related to systemic diseases, such as hypertension, diabetes, dyslipidemia, and obesity^16,17,22,26–29^. In these reports, the causative mechanisms of these relationships were suggested to be end-organ damage related to metabolic syndrome. Atherogenic dyslipidemia affects microvascular complications of the eyelid^28^, and blepharoptosis in diabetes is attributed to chronic tissue hypoxia of the levator muscle^30^. Reactive oxygen species resulting from metabolic syndrome may contribute to the degeneration of the eyelid^26^. Moreover, obesity and its related body composition indices, such as waist circumference (WC), fat mass (FM), fat mass index (FMI), BMI, and lean body mass index (LBMI), were related to the prevalence of blepharoptosis^16,17,26^. Fat gain on the upper eyelid due to obesity can cause mechanical push-down of the upper eyelid, stretching of the levator aponeurosis, and fatty inflammation^17^. In our study, multivariate analyses showed that MMI and sarcopenia were significantly associated with blepharoptosis, independent of confounding factors related to metabolic syndrome. In addition, our additional analysis using BMI supported this result and showed that blepharoptosis in men is strongly associated with sarcopenia compared to BMI. This implies that blepharoptosis in men is caused by a degenerative change in the levator muscle rather than by obesity related fat gain. Meanwhile, in women, obesity may influence the prevalence of blepharoptosis. This sex difference may be due to differences in sex hormones^30,31^.

Age particularly may interact with MMI and blepharoptosis among the confounding variables, further complicating the interpretation of the association between them. As getting older, MMI decreases, and the incidence of blepharoptosis increases (Table 1)^27,32,33^. Our stratified analyses into a middle-aged group (40–59 years) and an older-aged group (60–79 years) showed a consistent negative correlation between MMI and blepharoptosis in both groups. These results suggest the correlation between sarcopenia itself and blepharoptosis, independently from the effect of aging.

Many efforts and methodologies have been made to define sarcopenia^34,35^. A recent trend is to focus on muscle strength; according to the new definition in 2018^7^, low muscle strength is the primary parameter for “probable sarcopenia”, and low muscle quantity or quality can confirm the diagnosis. However, the KNEHS 2008–2011 study design did not include the assessment of muscle strength, such as the handgrip test. Therefore, in this study, we used MMI to apply ASM to quantify the muscle mass and define sarcopenia. Adjusting body size to ASM using BMI was introduced by the Foundation for the National Institutes of Health (FNIH) Sarcopenia Project in 2014^36^ and has been proven to be a better way to define sarcopenia in the Korean population and validated in multiple studies^37–39^. We defined sarcopenia as the lowest quintile of MMI not following the cut-offs of FNIH Sarcopenia Project suggested since the difference of the participants’ race and age distribution^38,40^. However, our cutoff values, 0.809 in men and 0.532 in women, are comparable to the cut-offs of FNIH Sarcopenia Project in 2014, which were 0.789 and 0.512 in men and women respectively.

Additionally, blepharoptosis may have the potential to be used as a screening tool to suggest sarcopenia. As shown in Table 1, participants with blepharoptosis were approximately three times more likely to belong to the lowest MMI quintile group (Q1, 29.2% in men, 30.8% in women) than those in the highest quintile group (Q5, 11.7% in men, 12.3% in women). In addition, as shown in Fig. 2, as the muscle mass index decreased, levator muscle function decreased, indicating reduced levator muscle strength. As discussed above, the relationship between blepharoptosis and MMI should be due to the systemic sarcopenic process, and blepharoptosis can be a practical screening tool for sarcopenia. Since SARC-F, sarcopenia screening questionnaire, and assessment of skeletal muscle strength are limited to handgrip test, chair stand test, and walking ability, blepharoptosis will be more meaningful when assessing patients with disabilities, such as quadriplegia, or selecting patients who should assess sarcopenia even before SARC-F screening.

This study has some limitations. First, it was a cross-sectional study and could not clarify causality. We could not explore the direct relationship between blepharoptosis and systemic muscle strength, such as in the handgrip test, because of limited data. However, the levator function represented the levator palpebrae superioris muscle strength, which could complement systemic muscle strength. Second, the data are restricted to the Korean population. The eyelid anatomical structures like orbital fat or insertion of levator aponeurosis may differ from other ethnicities; therefore, caution is needed when applying the results of this study to different ethnicities, and further research encompassing other ethnicities is valuable.

This is the first study to investigate the association between blepharoptosis and sarcopenia. Multivariate analysis revealed that sarcopenia and MMI were independently associated with senile blepharoptosis. Our results may explain the reduced levator muscle function induced by sarcopenic processes, such as myosteatosis, resulting in senile blepharoptosis. Our study is valuable as it shows that sarcopenia may impair an individual’s life by impairing visual function and aesthetics, and it provides a new perspective on the mechanisms of senile blepharoptosis.

## Methods

### Study population

All analyses were conducted using data obtained from the Korea National Health and Nutrition Examination Survey (KNHANES) between 2008 and 2011. This nationwide survey is a population-based, cross-sectional health examination and survey regularly conducted by the Korean Center for Disease Control and Prevention in the Ministry of Health and Welfare to monitor the general health and nutritional status of South Koreans. It comprises standardized health examinations and questionnaires on nutrition, lifestyle, and medical information. Of the 37,753 subjects from the KNHANES 2008–2011, we initially selected 20,748 patients who completed the ophthalmological examination and dual-energy X-ray absorptiometry (DXA, QDR 4500A, Hologic Inc., Waltham, MA). Among the participants aged from 10 to 80 years old, patients aged 40–79 years (n = 12,338) were included^32,33^. Those with anophthalmos (n = 22), facial palsy (n = 35), and thyroid diseases (n = 605), which could affect eyelid position, were excluded. Finally, 11,533 patients (5159 men and 6394 women) were included in the final statistical analysis.

The study was conducted in accordance with the ethical standards laid down in the 1964 Declaration of Helsinki and its later amendments. Written informed consent was obtained from all participants before the study began, and the KNHANES was conducted following ethical approval by the Institutional Review Board of the Korea Center for Disease Control and Prevention (2008-04EXP-01-C, 2009-01CON-03-2C, 2010-02CON-21-C, 2011-02CON-06C).

### Blepharoptosis and ophthalmic measurement

Trained ophthalmologists performed ophthalmic examinations. The participants underwent a comprehensive ophthalmic examination, including visual acuity measurement, automated refraction, slit-lamp biomicroscopy, intraocular pressure measurement, and fundus photography. Marginal reflex distance 1 (MRD1) was measured from the upper eyelid margin to the corneal light reflex after the participants looked straight at a distant target. We selected a subject eye with a smaller MRD1 of either eye, and defined blepharoptosis when the MRD1 of the eye was less than 2 mm. The levator function was measured by having the patient look down and with a hand on the individual’s forehead to prevent any brow action, asking the patient to look upward as far as possible without changing the head position. Elevation of the upper lid margin (in mm) is the levator muscle function. If the distance was over 8 mm, the participant was considered to have fair to good levator function. Slit-lamp examination confirmed lens status with cataract or intraocular lens. Individuals who were pseudophakic or aphakic were considered to have a history of cataract surgery.

### Sarcopenia and systemic assessment

In this study, we used the BMI-ASM definition as muscle mass index (MMI, ASM [kg] divided by BMI [kg/m^2^]) to quantify muscle mass and assess sarcopenia. Individuals were separated by sex and classified into quintiles of the MMI; those in quintile 1 (Q1), representing 20% of the patients with the lowest MMI, were considered to have sarcopenia. ASM, FM and LBM were measured using DXA^15^.

Participants who had systolic blood pressure ≥ 140 mmHg, diastolic blood pressure ≥ 90 mmHg, or had taken hypertension medication were included in the study. Diabetes was diagnosed in those with a fasting blood glucose level of ≥ 126 mg/dL or those taking diabetes medication or insulin. Dyslipidemia was defined as fasting blood total cholesterol > 240 mg/dL, triglyceride > 200 mg/dL, or taking lipid-lowering medication. Based on a self-administered questionnaire, a diagnostic history of stroke and ischemic heart diseases, including myocardial infarction and angina, was reported. Participants were assessed for their current smoking status. Regular exercise was considered vigorous exercise for more than 20 min, 3 days per week.

### Statistical analyses

A chi-square test was used to compare the clinical characteristics between the groups with and without blepharoptosis. The Cochran-Armitage test was performed to analyze the trend in the prevalence of blepharoptosis according to the MMI quintiles. Multivariate logistic regression analysis was performed to determine the relationship between MMI, sarcopenia, and blepharoptosis. Model 1 was adjusted for age, and model 2 was adjusted for hypertension, diabetes, smoking, and previous cataract surgery in addition to age. Obesity (BMI > 25 kg/m^2^) was added to model 3 for adjustment. R ver. 4.0.3. for Windows (The R Foundation for Statistical Computing, Vienna, Austria) was used for data analysis, and a p-value less than 0.05 was considered statistically significant. Continuous numeric data are represented as mean ± standard deviation.

## Supplementary Information

Supplementary Figures.

## Data Availability

The datasets generated during and/or analyzed during the current study are available from the corresponding author on reasonable request.
