# Supplementary material for: Sarcopenia as a potential risk factor for senile blepharoptosis: Nationwide Surveys (KNHANES 2008–2011)
Source: Sci Rep. 2023 Mar 29;13:5150. doi: 10.1038/s41598-023-31097-7 (PMC10060385; doi:10.1038/s41598-023-31097-7)
Supplement: Supplementary file 1 — Supplementary Figures. [file 41598_2023_31097_MOESM1_ESM.pdf]

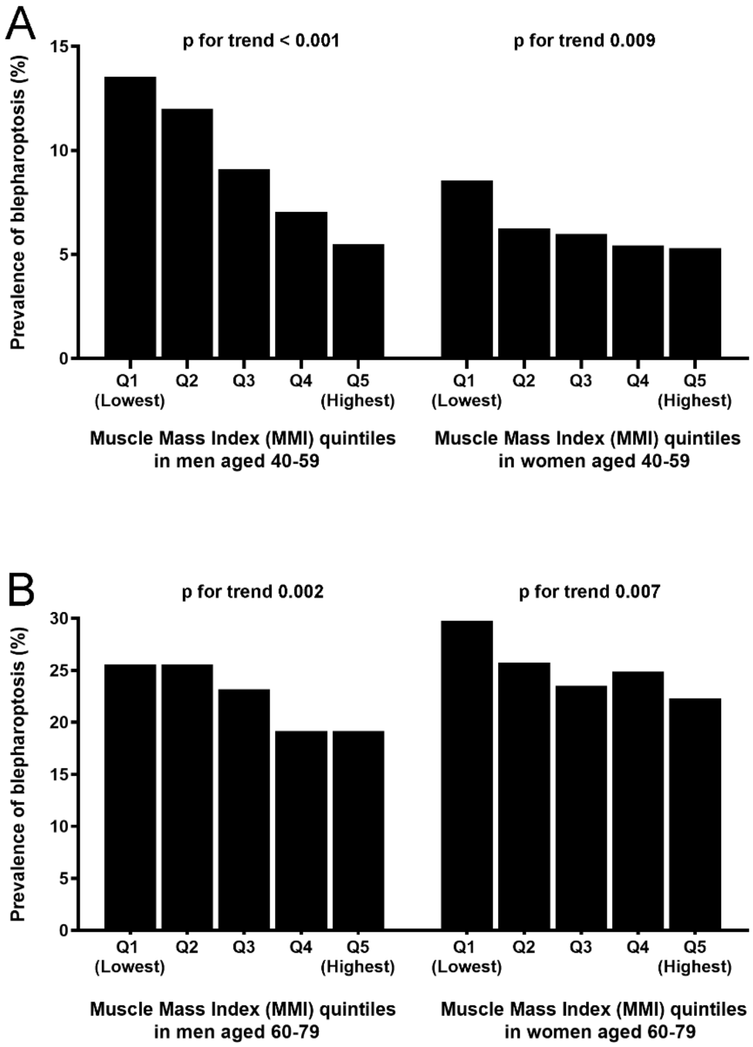

Supplementary Figure S1. Age-stratified analysis of MMI and prevalence of blepharoptosis. (A) In a group aged 40-59, MMI and blepharoptosis prevalence have a negative correlation (*p* value for linear trend < 0.001 in men, 0.009 in women). (B) The elder group aged 60-79 also showed a negative correlation between MMI and blepharoptosis prevalence in both men and women (*p* value for linear trend = 0.002 in men, 0.007 in women).

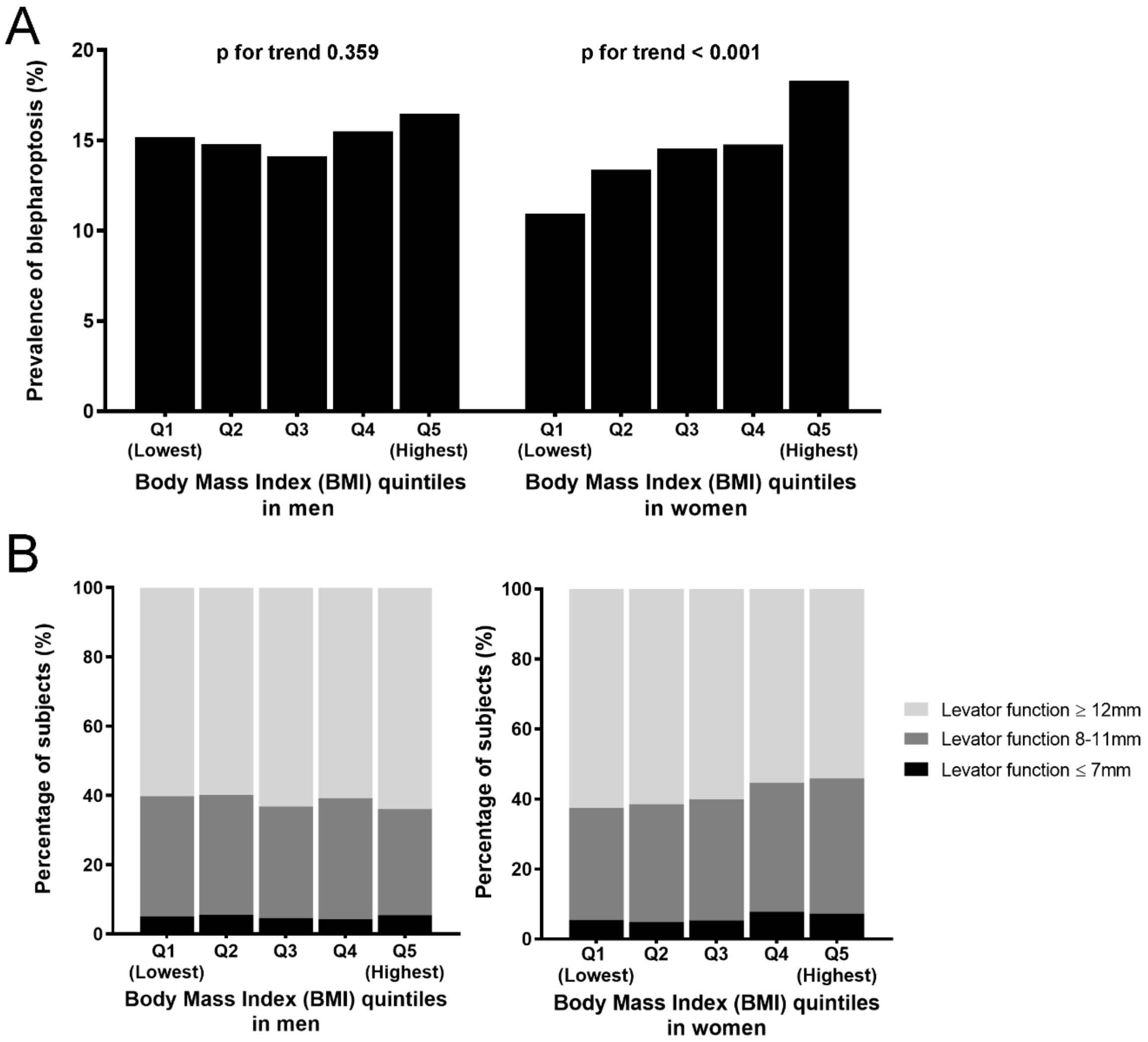

Supplementary Figure S2. BMI and prevalence of blepharoptosis with stratified with levator function. (A) The prevalence of blepharoptosis and BMI showed a positive relationship in women (p for trend < 0.001), but no such correlation was found in men (p value for linear trend = 0.359). (B) Similarly, in the analysis of levator function, there was a positive correlation between BMI and blepharoptosis prevalence in women (all p values for linear trend < 0.05), but not in men (all p values for linear trend > 0.05).
